# Serum ferritin levels and the development of metabolic syndrome and its components: a 6.5-year follow-up study

**DOI:** 10.1186/1758-5996-6-114

**Published:** 2014-10-26

**Authors:** Päivi Hämäläinen, Juha Saltevo, Hannu Kautiainen, Pekka Mäntyselkä, Mauno Vanhala

**Affiliations:** Department of Internal Medicine, Tampere University Hospital, Teiskontie 35, 33521 Tampere, Finland; Department of Internal Medicine, Central Finland Central Hospital, Jyväskylä, Finland; Unit of Family Practice, Central Finland Central Hospital, Jyväskylä, Finland; Unit of Primary Health Care, University of Eastern Finland, Kuopio, Finland; Unit of Primary Health Care, Kuopio University Hospital, Kuopio, Finland

**Keywords:** Metabolic syndrome, Ferritin, Obesity

## Abstract

**Background:**

The aim of this study was to investigate the relationship between changes in serum ferritin concentrations and the development of metabolic syndrome (MetS) and its components over a 6.5 year follow-up period in Finnish adults.

**Methods:**

Adults born in Pieksämäki, Finland, in 1942, 1947, 1952, 1957, and 1962 (n = 1294) were invited to health checkups between 1997 and 1998 and 2003 and 2004. All of the required variables for both checkups were available from 691 (53%) subjects (289 men and 402 women). MetS was defined by the National Cholesterol Education Program criteria.

**Results:**

During the 6.5-year follow-up period, 122 (18%) subjects developed incident cases of MetS. Increases in serum ferritin levels were significantly higher in both women and men with incident MetS compared with women and men without MetS (p = 0.04, p = 0.03). Also, serum ferritin levels increased significantly less in women in whom the criteria for MetS resolved during the follow-up period (p = 0.01). Increases in serum ferritin levels were significantly lower in women in whom the glucose criterion for MetS resolved, and higher in women for whom the waist criterion developed (p = 0.01 and p <0.001, respectively). Serum ferritin levels decreased significantly more in men in whom the triglyceride criterion for MetS resolved during the follow-up period (p = 0.01). There was a clear and significant correlation between change in serum ferritin level and change in waist circumference both in men and women (p <0.001, p <0.01). In addition, correlations between change in serum ferritin level and change in plasma triglyceride as well as glucose levels were strongly positive in men (p <0.001). There was negative correlation between change in serum ferritin and plasma high density cholesterol level both in men and women.

**Conclusions:**

Increases in serum ferritin over a 6,5 year period are associated with development of MetS in both men and women. Whereas, lower increases in serum ferritin over the same timeframe are associated with resolution of hypertriglyceridemia in men and hyperglycemia in women. Increases in waist circumference was positively correlated with increases in serum ferritin in both men and women.

## Background

Metabolic syndrome (MetS) is a pathophysiological disorder with a clustering of risk factors for cardiovascular disease and type 2 diabetes [1, 2]. Ferritin, an intracellular protein and key regulator of iron homeostasis, is a clinical measure of body iron stores [3]. Elevated body iron stores could promote oxidative stress, and in this manner affect the pathogenesis of insulin resistance [4–6]. Several studies have reported an association between elevated serum ferritin levels and elevated serum insulin, fasting glucose, insulin resistance [7–9], and diabetes [10–16]. Cross-sectional studies have found an association between metabolic syndrome (MetS) and serum ferritin levels [17–23]. No studies have been done to investigate the relationships between changes in serum ferritin levels and development of MetS components in both men and women. One prospective study previously evaluated an association between baseline serum ferritin levels and future MetS [24].

The aim of this population-based study was to investigate the relationship between changes in serum ferritin over a 6.5 year period and the development and resolution of MetS and its components in Finnish adults.

## Methods

All residents of Pieksämäki, a town in Finland, who were born in 1942, 1947, 1952, 1957, and 1962 (n = 1,294) were invited to receive a health checkup between 1997 and 1998 (baseline visit) and again between 2003 and 2004. Of those invited, 923 (71%) participated in the first checkup, and 693 subjects (54%) attended both checkups. All variables analyzed in the present study were available from 691 subjects (289 men and 402 women). All the subjects completed a questionnaire that asked about their medications, smoking habits, alcohol consumption, and level of physical activity. The protocol was approved by the Kuopio University Hospital Ethics Committee. All participants provided written informed consent.

### Clinical and laboratory measurements

Health evaluations were performed by the same 2 nurses at both checkups. Sitting blood pressure was measured with a mercury sphygmomanometer after 15 minutes of rest. The measurement was repeated 5 minutes later, and the mean of the 2 measurements was used in the statistical analyses. Waist circumference was measured from the midpoint between the lateral iliac crest and the lowest rib to the nearest 0.5 cm. Weight and height were measured to the nearest 0.1 kg and 0.5 cm, respectively.

Blood samples were taken after an overnight fast. Plasma was separated by centrifugation and the samples were frozen immediately and stored at -70C. Samples were analyzed in Kuopio regional laboratory during the year 2010.

Plasma glucose concentration was measured using an automated colorimetric method (Peridochrom Glucose GOD-PAP; Boehringer Mannheim GmbH, Mannheim, Germany). Serum triglycerides and cholesterol were measured from fresh serum samples using glycerol-3-phosphate oxidase phenol + aminophenazone (PAP) and cholesterol oxidase-PAP (CHOD-PAP) enzymatic colorimetric methods, respectively (Boehringer Mannheim GmbH). Serum high-density lipoprotein (HDL) cholesterol was measured using the same method (CHOD-PAP) after the precipitation of apolipoprotein B-containing lipoprotein particles by phosphotungstic acid and magnesium. High-sensitivity C-reactive protein (hs-CRP) was measured with an Immulite® analyzer and a DPC high-sensitivity CRP assay (Diagnostics Products Corporation, Los Angeles, CA, USA). Serum ferritin concentration was analyzed using an electrochemiluminescence immunoassay (Roche Diagnostics GmbH, Mannheim, Germany). In order to exclude liver storage disease plasma alanine aminotransferase (P-ALT) was measured using a kinetic method according to the International Federation of Clinical Chemistry and Laboratory Medicine using a cobas® 6000 (c 501) analyzer (Hitachi High Technology Co, Tokyo, Japan).

At the beginning and end of the study period, MetS was defined according to the new harmonized criteria [23]. Subjects with 3 or more of the following components were classified as having metabolic syndrome: 1) waist circumference ≥102 cm for men and ≥88 cm for women 2) fasting triglycerides ≥1.7 mmol/L or treatment for dyslipidemia 3) serum HDL cholester <1.03 mmol/L for men and <1.29 mmol/L for women or treatment for dyslipidemia 4) systolic blood pressure ≥130 mmHg or diastolic blood pressure ≥85 mmHg or the use of antihypertensive medication 5) fasting plasma glucose of ≥5.6 mmol/L or the use of medication for hyperglycemia.

### Statistical analyses

The results are expressed as means and standard deviations (SDs) for continuous variables and as proportions for categorical variables. The normality of variables was evaluated by the Shapiro-Wilk W-test. Statistical comparisons between the groups were performed using the chi-square test, t-test, or bootstrap-type t-test as appropriate. Bootstrap type analysis of covariance was also used to compare the groups as measurements. In these analyses, the baseline variables of age, smoking, physical activity, serum ferritin levels, body mass index and hs-CRP were used as covariates. Partial correlations were calculated between chance in serum ferritin level and chance in levels of MetS components and adjusted for age, and baseline smoking, physical activity, alcohol use, serum ferritin concentration, body mass index, and hs-CRP. For all analyses, p <0.05 was considered significant.

## Results

All variables from both checkups were available for 691 subjects (289 men and 402 women). The baseline characteristics of the study participants are presented in Table 1. At baseline, MetS was present in 31% of the subjects. During the follow-up time, 122 (18%) incident cases of MetS developed and 44 (6%) cases of MetS resolved (data not shown).

**Figure 1:**
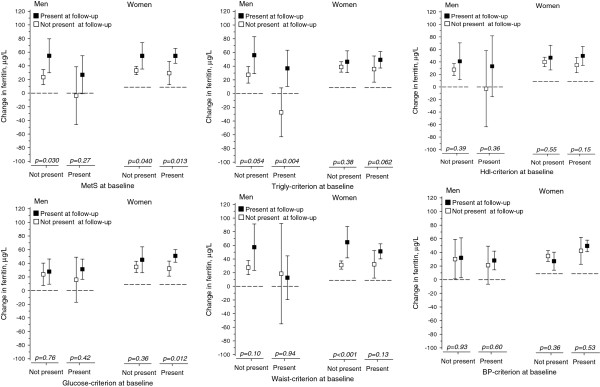
**Legends: All results are adjusted for age, baseline smoking, baseline physical activity, baseline use of alcohol, baseline serum ferritin level, baseline body mass index and baseline hs-CRP.** Waist criterion = Waist >102 cm (male) or >88 cm (female), Glucose criterion = FP-glucose ≥5.6 mmol/L, Trigly criterion = Triglycerides >1.7 mmol/l or medication for dyslipidemia, HDL criterion = HDL–cholesterol <1.03 mmol/l (men) or <1.29 mmol/l (women) or medication for dyslipidemia, BP criterion = Systolic blood pressure ≥130 mmHg or diastolic ≥85 mmHg or antihypertensive medication.

Development of MetS and MetS components in relation to changes in serum ferritin levels during the follow-up period are shown in Figure 1. All results were adjusted for the baseline variables of age, smoking, physical activity, use of alcohol, serum ferritin level, body mass index and hs-CRP. Serum ferritin level was significantly higher both in women and men with incident MetS compared with women and men without MetS (p = 0.04, p = 0.03). Serum ferritin levels increased significantly less in women in whom the criteria for MetS resolved during the follow-up period compared with women in whom the MetS criteria remained (p = 0.01). Increases in serum ferritin levels were significantly lower in women in whom the glucose criterion of MetS resolved during the follow-up period compared with women in whom the glucose criterion remained (p = 0.01). Serum ferritin levels decreased significantly more in mean whom the MetS triglyceride criteria for triglycerides resolved during the follow-up period compared to men who continued to meet the MetS criteria for triglycerides (p = 0.004). Also, there was a statistical trend suggesting an increase in serum ferritin over the follow-up period was associated with the development of hypertriglyceridemia compared to men who did not develop hypertriglyceridemia (p = 0.05). Increases in serum ferritin were significantly associated with increases in waist circumference in women during the follow-up period (p = 0.0001). Changes in ferritin levels between subjects whose HDL or blood pressure criterion developed or resolved during follow-up time did not differ significantly.

**Table 1:** **Baseline clinical and life-style characteristics of the study population**

Characteristics	Men N = 289 (42%)	Women N = 402 (58%)	All N = 691
MetS criteria present, n (%)	98 (34)	116 (29)	214 (31)
Age, years, mean (SD)	45.3 (6.2)	45.1 (6.5)	45.2 (6.2)
BMI, mean (SD)	26.8 (3.5)	26.3 (5.2)	26.6 (4.6)
Waist, cm, mean (SD)	94.2 (10.2)	83.4 (12.3)	87.9 (12.6)
FP-glucose mmol/L, mean (SD)	5.9 (0.9)	5.6 (0.6)	5.8 (0.8)
BP systolic, mmHg, mean (SD)	137.7 (16.5)	132.2 (18.2)	134.4 (0.7)
BP diastolic, mmHg, mean (SD)	83.5 (9.7)	79.4 (9.5)	81.1 (9.8)
HDL-C, mmol/L, mean (SD)	1.3 (0.3)	1.5 (0.3)	1.4 (0.3)
Triglycerides, mmol/L, mean (SD)	1.7 (1.3)	1.2 (0.6)	1.4 (1.0)
ALT (U/L) mean (SD)	18.0 (10.9)	12.0 (7.6)	14.5 (9.6)
Hs-CRP (mg/L), mean (SD)	1.7 (3.9)	1.7 (2.3)	1.7 (3.0)
Life-style factors, n (%)			
Current smoker	80 (28)	82 (20)	162 (23)
Current use of alcohol			
Low (nothing)	36 (12)	85 (21)	121 (18)
Moderate	167 (58)	290 (72)	457 (66)
High	86 (30)	27 (7)	113 (16)
Physical activity n (%):			
Low	41 (14)	54 (13)	95 (14)
Moderate	161 (56)	240 (60)	401 (58)
High	87 (30)	108 (27)	195 (28)

BMI: Body mass index; FP-glucose: fasting plasma glucose; BP systolic: systolic blood pressure; BP diastolic: diastolic blood pressure; HDL-C: high density cholesterol; Hs-CRP: high sensitivity C-reactive protein; ALT: alanine aminotransferase.

Current use of alcohol was considered low with no use of alcohol, moderate with use of <2 portions/day, and high with use of >2 portions/day. Physical activity was considered to be high in subjects who exercised at least 30 minutes daily in their leisure time, moderate in subjects who exercised at least three times per week, and low if exercising frequency was less than three times per week.

Partial correlations between change in serum ferritin level and change in levels of MetS components are shown in Table 2. All results are adjusted for the baseline variables of age, smoking, physical activity, use of alcohol, serum ferritin level, body mass index and hs-CRP. There was strong positive correlation between change in serum ferritin level and change in waist circumference both in men and women (p <0.001, p <0.01). In addition, correlations between change in serum ferritin level and change in plasma triglyceride as well as glucose levels were strongly positive in men (p <0.001). There was negative correlation between change in serum ferritin and change in plasma HDL cholesterol level both in men and women. The correlations between change in serum ferritin level and change in systolic or diastolic blood pressure level were not significant.

**Table 2:** **Partial correlations between change in serum ferritin level and change in levels of MetS components**

	Men r (95% CI)	Women r (95% CI)
**Waist**	0.21 (0.11 to 0.35)***	0.15 (0.05 to 0.24)**
**Triglycerides**	0.20 (0.11 to 0.31)***	0.09 (-0.01 to 0.17)
**HDL-C**	-0.12 (-0.25 to -0.01)*	-0.13 (-0.22 to -0.02)*
**Glucose**	0.20 (0.07 to 0.33)***	0.08 (-0.02 to 0.18)
**Systolic BP**	-0.03 (-0.15 to 0.11)	0.02 (-0.07 to 0.12)
**Diastolic BP**	0.03 (-0.11 to 0.18)	0.03 (-0.09 to 0.13)

*p <0.05, **p <0.01, ***p <0.001.

Adjusted for age, baseline smoking, baseline physical activity, baseline use of alcohol, baseline serum ferritin level, baseline body mass index and baseline high sensitivity C-reactive protein.

HDL: high density cholesterol; Systolic BP: systolic blood pressure; Diastolic BP:diastolic blood pressure.

## Discussion

To the best of our knowledge, this is the first follow-up study evaluating changes in serum ferritin levels in relationship with the development and resolution of MetS and components of the MetS in Finnish men and women.

The increase in serum ferritin levels during the follow-up period was significantly higher in men and in women with incident MetS compared with men and women without incident MetS. In addition, reductions in ferritin levels were significantly higher in women in whom the criteria of MetS resolved during the follow-up period. Previous longitudinal studies have evaluated changes in ferritin levels and MetS [17, 18, 20, 21, 23, 25–27]. Also, a recent longitudinal study showed that elevated ferritin levels at baseline are associated with future development of MetS in Korean men [24]. Our study shows not only an association between baseline increased ferritin levels and future MetS, but also between increased or reduced ferritin levels and MetS development or resolution, respectively, during the follow-up period. We also show an association in both sexes and extended our evaluation to all MetS components. No previous longitudinal studies have been done to evaluate the changes in ferritin levels and development of all MetS components.

We show that serum ferritin was significantly higher or trended towards being higher in men with the triglyceride criterion for MetS resolved or developed over the follow-up period. We also show strong positive association between change in serum ferritin level and triglyceride level in men. This agrees with previous cross-sectional studies that found an increasing prevalence of elevated triglycerides with increasing serum ferritin levels [20, 26]. Other studies showed that higher ferritin concentrations were associated with increased triglyceride concentrations [23, 27]. Also, higher ferritin concentrations were previously found to be associated with an increase in triglyceride levels in male patients with iron overload and homozygosity for human hemochromatosis gene mutations [28].

We show that serum ferritin levels increased significantly less in women in whom the glucose criterion for MetS resolved during the follow up period and also a strong positive association between change in serum ferritin level and glucose level in men. Several longitudinal studies have previously shown an association between incident diabetes and higher baseline ferritin levels [10, 12–16, 29].

Serum ferritin levels increased significantly more in women whose waist criterion for MetS developed during the follow up period. Also, there was a strong positive correlation between change in serum ferritin level and change in waist circumference both in men and women. Waist circumference was the only one of the Mets components that was positively associated with change in ferritin level both in men and women, which may indicate the importance of waist circumference in development of Mets. Our results are in line with previous studies that found an association between serum ferritin levels and central adiposity [30] or an increasing prevalence of the MetS waist criterion with increasing serum ferritin levels [23, 26]. However, parts of the previous cross-sectional studies found no association between the MetS waist criterion and ferritin levels [27].

The mechanism underlying serum ferritin levels and the development of MetS is not established, but iron accumulation and oxidative stress is the leading hypothesis. Iron is involved in multiple cellular processes and is important for the activity of various enzymes, but it can also be toxic and cause organic biomolecular oxidation [31]. Ferritin is a clinical measure of body iron stores [3]. Elevated body iron stores may promote oxidative stress, that may contribute to cellular damage leading to insulin dysfunction, insulin resistance and abnormal pancreatic beta-cell function [2, 4–6, 32]. Hepatic iron overload has been shown to contribute to peripheral hyperinsulinemia and insulin resistance, while muscular iron accumulation contributes to decreased glucose utilization [33]. Moreover, phlebotomy with a moderate reduction in body iron stores measured by serum ferritin levels resulted in improvements in glycemic control in patients with MetS in a controlled clinical trial [34]. Chronic oxidative stress is also associated with oxidation dysfunction of long chain fatty acids in mitochondria, which can lead to hypertriglyceridemia in circulation and excessive triglyceride accumulation in muscle and liver tissue [35, 36]. These mechanisms support our findings that increasing ferritin levels predict incident MetS and hyperglycemia and hypertriglyceridemia components of MetS.

The iron-regulatory hormone hepcidin control the dietary absorption, storage, and tissue distribution of iron. When hepcidin concentrations are high, iron is trapped in enterocytes, macrophages, and hepatocytes [37]. Adipose tissue expressed hepcidin has shown to be enhanced in obese patients [38]. It is possible, that when waist circumference and central adiposity increase, also hepcidin expression increases, and altered iron homeostasis leads to a change in ferritin levels.

Because ferritin is also an acute phase reactant, all results in our study were adjusted for hs-CRP levels to estimate the impact of inflammation. Results remained significant after adjustment that can suggest that ferritin concentrations are reflective of storage iron and not the acute phase response.

Some limitations of our study should be mentioned. We were not able to exclude persons with known *HFE* gene mutations. However, there was no need to exclude subjects with high baseline serum ferritin levels, considering that all baseline ferritin levels were less than 750 μg/l (range, 2–722 μg/l). Also, all baseline plasma ALT levels were less than 120 U/L (range, 4-120 U/L) that does not refer liver storage disease. Unfortunately, information about the female participant’s menopausal status or possible change in status during the follow up period was not available. Mean ferritin levels are known to be higher in premenopausal women than in postmenopausal ones [26, 39]. Also, information about the nutritional content of subjects’ diets or their consumption of dietary supplements like iron or antioxidants were not available. The relatively small number of MetS cases that resolved during the follow up period is also a limitation, and could have affected the non-significant results observed in men. However, the longitudinal, population-based design is the strength of this study.

In conclusion, serum ferritin level works as a follow-up and risk assessment measure in subjects with metabolic risk factors; increasing levels indicating developing MetS and decreasing levels indicating resolving hypertriglyceridemia and hyperglycemia.
